# X-ray Reflectivity Study of Polylysine Adsorption on the Surface of DMPS Monolayers

**DOI:** 10.3390/membranes12121223

**Published:** 2022-12-02

**Authors:** Aleksey M. Tikhonov, Victor E. Asadchikov, Yury O. Volkov, Boris S. Roshchin, Alexander D. Nuzhdin, Kirill I. Makrinsky, Yury A. Ermakov

**Affiliations:** 1 Kapitza Institute for Physical Problems, Russian Academy of Sciences, Moscow 119334, Russia; 2 Shubnikov Institute of Crystallography, Federal Research Center Crystallography and Photonics, Russian Academy of Sciences, Moscow 119333, Russia; 3 Institute of Solid State Physics, Russian Academy of Sciences, Chernogolovka 142432, Russia; 4 Frumkin Institute of Physical Chemistry and Electrochemistry, Russian Academy of Sciences, Moscow 119071, Russia

**Keywords:** phase transition, Langmuir monolayers, compressibility, Volta potential, lipid hydration, polylysine adsorption, synchrotron X-ray reflectometry, polymer films, electron density profile

## Abstract

The results of a systematic study on the adsorption of polylysine molecules of different lengths on the surface of a 1,2-dimyristoyl-*sn*-glycero-3-phospho-L-serine (DMPS) monolayer in the liquid (LE) and condensed (LC) states are presented. A compressibility diagram and the Volta potential were recorded with the Langmuir monolayer technique and further analyzed with the empirical approach. The structure of the monolayer films with adsorbed polypeptides was studied with synchrotron X-ray reflectometry. Two- and three-layer slab models describe the reflectivity data fairly well and reveal both the significant structural changes and the dehydration of the polar groups induced by all polylysines used at the maximal coverage of the monolayer interface in both the LE and LC states. On the one hand, in the LE phase of the monolayer (area per molecule A ≅ 70 Ǻ^2^), the integrated electron density of the lipid headgroup region is approximately half the density contained in the clean monolayer. This indicates both significant compaction and dehydration in the polar groups of the lipids, caused by the adsorption of polypeptides. On the other hand, in the LC state (A ≅ 40 Ǻ^2^), the degree of the hydration of the polar region is similar to that for the initial DMPS monolayer. However, both the electron density and the thickness of the head group region differ significantly from the values of these parameters for the clean monolayer in the LC state.

## 1. Introduction

The Langmuir layers of phospholipids have been used for many years as a model to study the surface characteristics of biomembranes, mostly determined using a lipid matrix in the environment of the membrane proteins. The focus of many experimental physicochemical studies is the interaction of positively charged polypeptides of high molecular weight with phospholipids, which are responsible for local membrane structures. Physical and chemical conditions related to their formation, as well as various biomedical applications associated with these objects, are the subject of a large number of original and review publications. Authors have emphasized the special role of the anionic components of membranes in their interactions with polypeptides [1,2,3] and discuss hydrophobic and electrostatic input on the macromolecular/lipid structure as a factor of lipid segregation in lipid monolayers as a model of natural membranes [4]. Synthetic lysine- and arginine-based polycations are suggested as the best object to study the physical motives of natural peptides at the membrane–water interface [5,6]. Many polypeptides are polycations and bear a significant positive charge, activating their interaction with cell membranes, which are usually negatively charged [7]. A special interest is directed at understanding the polycation effect in the transport phenomena of the membranes, realized by specific membrane proteins with ionic channels [8], which have recently been linked to the biocidal function of newly developed drugs [9,10]. In addition, we direct readers to a recent review [11] referring to a sufficient number of original studies of the molecular mechanisms involved in the hydrophobic and electrostatic interactions of macromolecules with the surfaces of lipid and cell membranes, which are most pronounced in the case of large polypeptides. However, the characteristics of the lipid–water interface in the presence of water-soluble polypeptides, which can affect both the electric charge distribution on the membrane surface and the structural organization of the polar region of the lipid matrix, remain much less studied.

Synthetic lysine-based polypeptides may serve as a convenient object in studies of their interactions with the surfaces of lipid monolayers, which, in principle, can manifest themselves in different ways depending on the size of the macromolecules and the presence of charged components in the membrane. Moreover, the phase state of anionic phospholipids in cell membranes has a direct impact on the transportation processes mediated by specific transportation systems and ion channels [8,12]. In our work, we focus on the behavior of the dimyristoyl derivatives of phosphatidylserine (DMPS) molecules, whose hydrocarbon chains exhibit a first-order phase transition between the states of “liquid expanded” (LE) and “condensed two-dimensional crystal” (LC). We recently succeeded in revealing the physical nature of the phase transition by measuring the reflectivity of a grazing X-ray beam, which allowed us to quantify the electron-density distribution along the normal to the monolayer surface in its liquid and condensed states [13]. Full atomic modeling of the DMPS monolayer with molecular dynamics methods shows the significant role of the hydration shell of the phospholipid polar groups in the LE-LC phase transition in the monolayer. We have some experience in studying the electrical phenomena associated with the adsorption of polylysines on the surface of liposomes and planar lipid membranes accessible to bioelectrochemical methods [14,15,16,17]. In addition, in previous studies, we were able to prove the effectiveness of X-ray reflectometry in studying their interaction with the monolayers of DMPS phospholipids [18,19,20].

X-ray reflectometry with the use of a bright synchrotron source is a traditional method of obtaining information about the structure of the macroscopically planar surface of a liquid [21], namely, in relation to determining the structures of various kinds of adsorption films [22] and studying the phase transitions in them [23]. This technique, together with the Langmuir method, was successfully used in the study of protein–membrane binding and aggregation [24,25]. This motivates us to apply the same approach to the study of polylysine layers on the surface of a monolayer, which, to a certain extent, models the structure of the interface between the membrane and the water-soluble natural polypeptide. Below, we present a set of our experimental data indicating significant structural changes in DMPS monolayers in both the LE and LC states in the presence of poly-D-lysine hydrobromide molecules at the interface with water. These data were obtained from measurements of the compressibility of the lipid monolayer using the Langmuir technique, as well as using synchrotron X-ray reflectometry.

## 2. Materials and Methods

Sodium salt, C_34_H_65_NO_10_PNa, of DMPS phospholipid (Avanti Polar Lipids, Birmingham, AL, USA) was prepared in a solution of a mixed composition of chloroform–methanol, 5:1, at a lipid concentration of 0.5 mg/mL. A 10 mM KCl solution in doubly distilled and deionized water (pH = 7) was used as the main subphase. The molecular weight of poly-D-lysine hydrobromide, [H_12_C_6_N_2_O]_n_ HBr (M.W. of a single base is 209), and the approximately estimated length of the polypeptides are presented in brackets according to the Sigma catalog (USA) under the numbers P7886 (PL-200), P0296 (PL-12), and L9151 (PL-5). Due to the presence of two amino groups at lysine bases (pK = 8.95 and 10.5) and one carboxyl group (pK = 2.18), they carry a single positive charge, and linear polymer molecules are charged in a wide pH range (5.0–9.0). The control measurement of pH, with lysine solutions up to a 1 M concentration in the absence of a buffer (10 mM KCl solution) does not reveal a significant change in pH.

**Figure 1:**
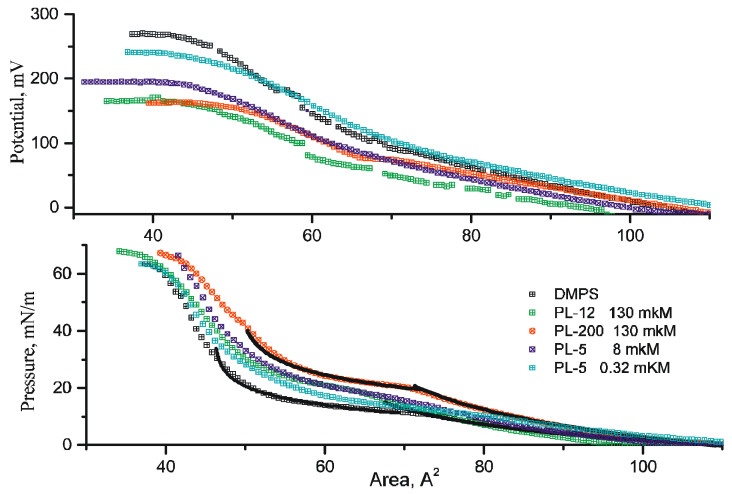
Volta potential (**upper panel**) and lateral pressure (**lower panel**) of DMPS monolayers at different areas of the lipid per molecule in the presence of polylysines of different lengths. The change in the Volta potential is shown for the region of continuous monolayers existing at pressures above 1 mN/m. The potential at 1 mN/m was assumed to be zero. The experimental points are sparse for convenience and the clarity of the image. The solid lines correspond to the simplest empirical description of the monolayer compression in the LE region with the parameters given in Table 1.

**Figure 2:**
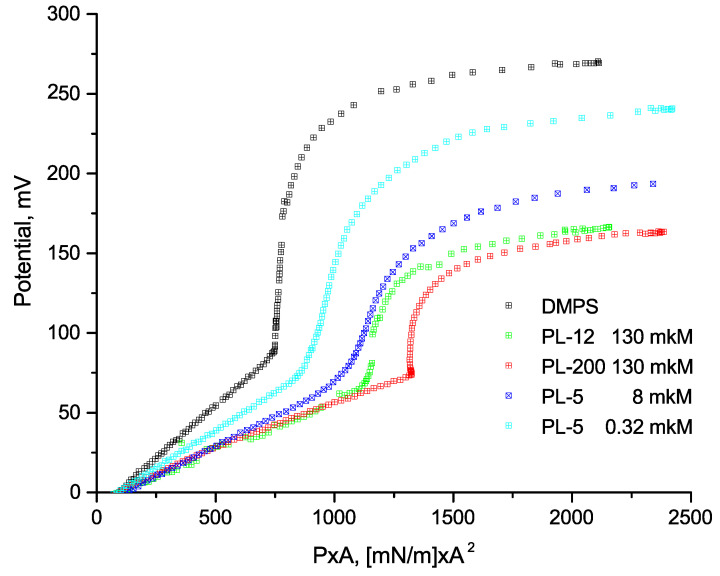
Changes in Volta potential measured for DMPS monolayers in the presence of polylysines as a function of the two-dimensional compression energy of the monolayer. For all curves, the zero value of the potential is taken at the compression point corresponding to the lateral pressure in the monolayer equal to 1 mN/m.

Pressure-area diagrams and boundary (Volta) potential were measured simultaneously on a MicroTrough XS V4.0 setup (Kibron Inc., Helsinki, Finland) with tetrafluoroethylene through a 20 × 6 cm opening (volume 75 mL), 2 barriers of polyoxymethylene, and a vibrating Kelvin electrode to record the boundary potential. Before each experiment, the surface pressure of the subphase with polypeptides in the absence of a lipid monolayer was controlled to be sure that no surface activity was detected up to a minimal distance between barriers. All measurements were carried out under normal conditions at room temperature 20 min after lipid application and at a compression rate of 10 mm/min. According to the previously obtained data on electrokinetic measurements [26], the maximum coverage of the surface of liposomes depends on the surface area available for adsorption. Therefore, in experiments on the compression of the lipid monolayer, the concentration of polylysines in the aqueous subphase was varied in order to estimate the amount sufficient to achieve the maximum effect. Figure 1 and Figure 2 depict the results for two pentalysine concentrations (PL-5). The corresponding amounts of polymers in the water subphase are provided in Figure 1 and Figure 2 in the units of polymer base concentration.

When performing X-ray experiments, a DMPS lipid monolayer was deposited in a sample cell with a volume of 36 mL on the surface of the subphase with a measuring syringe (Hamilton, Reno, NV, USA) in an amount corresponding to areas per lipid molecule of more than 70 Å^2^ (LE-phase) and near a collapse of about 40 Å^2^ (LC-phase). The polypeptides were introduced into an aqueous 10 mM solution of KCL in an amount equal to 0.5 mg (about 66 μM bases in the cell), which provided the maximal coverage of the lipid surface by the adsorbed polymer. Measurements of the X-ray reflection coefficient, *R*, for Langmuir DMPS monolayers at the water–air interface were carried out at the ID31 station of the European Synchrotron Radiation Source (Grenoble, France) [27]. The intensity of a focused monochromatic photon beam with a wavelength of ~0.175 Ǻ (the photon energy *E* ~71 keV, ΔE/E = 0.4%) was ~10^10^ f/s at the beam’s cross-sectional dimensions: ~6 μm in height and ~150 μm in the horizontal plane. The technique for measuring *R* is described in [18]. Samples of monolayers of phospholipid DMPS were prepared under conditions similar to those described above for measuring compression diagrams but in a round (10 cm in diameter) bath–plate made of tetrafluoroethylene. The latter was placed in a sealed thermostat with X-ray transparent windows [28].

## 3. Results

### 3.1. Compression of Langmuir Monolayers

Compression diagrams of a DMPS monolayer measured in the presence of polylysines of different molecular weights and lengths are shown in Figure 1. The shape of the curves clearly depends on the average area per lipid molecule, and their slope especially varied at the phase transition from the liquid LE to the condensed state LC. According to our electrokinetic data, published in [17], the adsorption of polylysines on the surface of negatively charged liposomes is accompanied by the neutralization of the surface, with a subsequent sharp increase in the positive surface (zeta) potential of liposomes up to saturation. At the saturation level, the range of the polymer contents in the suspension, expressed in terms of single base concentrations, significantly exceeds the amount of lipid in the suspension (about 1 mg/mL). Since the adsorption of polylysines is extremely efficient, the complete coverage of the surface by polymers depends on the size of the surface available for their adsorption, i.e., on the amount of lipid in the suspension [26]. The same results are well known for the adsorption of polycations of other chemical natures and different lengths [29]. Since the surface area of the monolayer is much smaller than the total surface area of the liposomes in suspension, the amount of polymer needed to coat the monolayer is much smaller. To estimate the proper concentration, we carried out measurements at different polymer concentrations in the subphase. Figure 1 and Figure 2 present data for two concentrations of pentalysine (PL-5), demonstrating that the shape of the experimental curves changed and shifted to similar curves measured for high-molecular-weight polylysines at their high concentration in the subphase. For large molecules, measurements at intermediate concentrations are not shown in the figures for clarity. When measuring the X-ray reflectance, solutions with high concentrations of polymers (>100 μM) were chosen, which, apparently, provide the maximum possible coverage of the monolayer surface. The corresponding compression diagrams for DMPS monolayers with PL-12 and PL-200 polymers are shown in Figure 1 and Figure 2.

An indirect factor indicating the alterations can be concluded from the above data by qualitatively analyzing the mechanochemical and electrical characteristics of Langmuir monolayers within the framework of the simplified empirical model, proposed previously in [30]. The area occupied by each lipid molecule in the monolayer depends on the lateral pressure in the monolayer, *P*, which is formally represented as a rigid “core”, *A_0_*, surrounded by an elastic shell with an area of *A_e_*. The size of the latter changes with compression pressure in accordance with its elasticity, determined by the parameter *K_p_*. A decrease in the value of parameter *K_p_* corresponds to an increase in the “rigidity” of the monolayer.

Figure 1 shows several theoretical curves with solid black lines, the parameters of which were found by fitting the experimental data with formula *A* = *A*_0_ + *A_e_* × exp(−*P/K_p_*), which is presented in Table 1. The small values of the auxiliary parameter, *t*_0_, reflect a good approximation of the theoretical curve of the experimental data. Coefficient *S* describes the linear relationship between the Volta potential and the work of monolayer compression in the region of its liquid LE state, which follows from the data in Figure 2.

Generally, the same formalism can be applied to describe the shape of curves at a pressure above the phase transition. It is not correct for the limited intermediate region where variable equilibrium occurs between the LE and LC blocks of lipid molecules (the fitting results are shown in Figure 1 with solid black lines). Obviously, this corresponds to a significant decrease in the compression work, which formally reflects the lower value of the compressibility modulus, *K_p_*. At the same time, the area per lipid decreases slightly at lower values for *A*_0_ and *A_e_*. It seems natural to attribute this fact to the dehydration of lipids. We do not believe that this assumption is indeed supported by this approach, but it is consistent with the results of the X-ray reflectivity data analysis.

According to our data (Figure 1), the slope of the Volta-potential drop at the lipid–water interface during the lipid phase transition in the presence of polylysines becomes significantly smaller than in the initial DMPS monolayer. This fact is quite consistent with the complex charge distribution at the interface. In a general case, the total alteration of the boundary (Volta) potential corresponds to the surface charge in the polycations that appeared at the interface, which is reflected in the surface potential, as well as in the dipole component of the boundary potential due to the reorientation of some dipole moments. We may conclude that the adsorption of polylysines affects both components of the boundary potential. At the same time, changes in the Volta potential due to the appearance of positive charges at the interfaces are compensated to some extent by the orientation of the dipoles at the interface in the opposite direction: its surface component becomes more positive, and the dipole component decreases. We drew the same conclusion in [14] from electrokinetic experiments on the adsorption of lysine molecules on negatively charged liposomes in combination with measurements of the boundary potential of planar lipid membranes under the same conditions. To understand the physical reasons for this phenomenon, a lipid–lysine system was simulated and analyzed via molecular dynamics methods. Indeed, it was found in [14] that the presence of adsorbed lysine molecules affects the orientation of water molecules and the network of hydrogen bonds in the polar region of the lipid bilayer. It induces a significant alteration in the network of lateral hydrogen bonds with the phosphate group of the phospholipid. It is natural to assume that the adsorption of large polylysine molecules can be accompanied by similar phenomena.

The recalculation of the experimental data for the Volta potential into the two-dimensional energy of the compressed monolayer, shown in Figure 2 makes it possible to more clearly identify the main factors affecting the interfacial potential. According to our data, the changes in the Volta potential in the region of the liquid LE state are directly proportional to the variation in the monolayer energy, with good accuracy. For comparison, Table 1 shows the slope factor, S, of the two compression curves in the LE region, reflecting a significant decrease in the interfacial potential in the presence of the polymer. Near the phase transition point, the difference between the surface potential of the clean DMPS monolayer and the potential of the monolayer in the presence of the adsorbed polymer is about 50 mV.

Earlier, in [14], we found approximately the same potential difference as a result of the adsorption of these polymers when comparing the surface potential of liposomes (data on electrokinetic measurements) with the boundary potential of planar BLMs (data on the compensation of the intramembrane field). Note that the changes in the boundary and Volta potentials of the monolayers have the same physical nature and, therefore, are equivalent. The results of modeling the lysine–lipid system presented in the cited work indicate a possible reason for the abovementioned effect, which is due to the contribution of lysine molecules to the change in hydrogen bonds in the polar region of the membrane. This phenomenon convinces us that the adsorption of polylysines on the monolayer surface can be accompanied by similar processes mediated by changes in the hydration state of the phospholipid.

### 3.2. X-ray Reflectometry Data and Analysis

**Figure 3:**
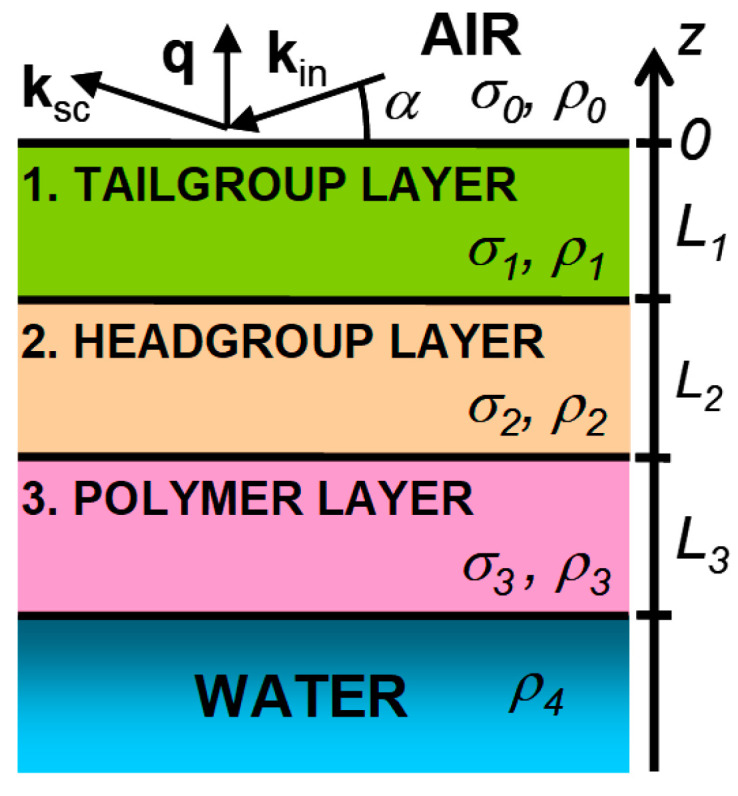
Cartoon for multilayer model and its DMPS parameter (layers 1 and 2) and adsorbed polymer films (layer 3).

Let **k**_in_ and **k**_sc_ be wave vectors with an amplitude of *k*_0_ = 2*π/λ* for the incident and scattered beams, respectively. It is convenient to introduce a coordinate system in which the origin, *O*, lies at the center of the illumination region, the *XY*-plane coincides with the water boundary, the *Ox* axis is perpendicular to the beam direction, and the *Oz* axis is directed along the normal to the surface opposite to gravity (see Figure 3).

**Figure 4:**
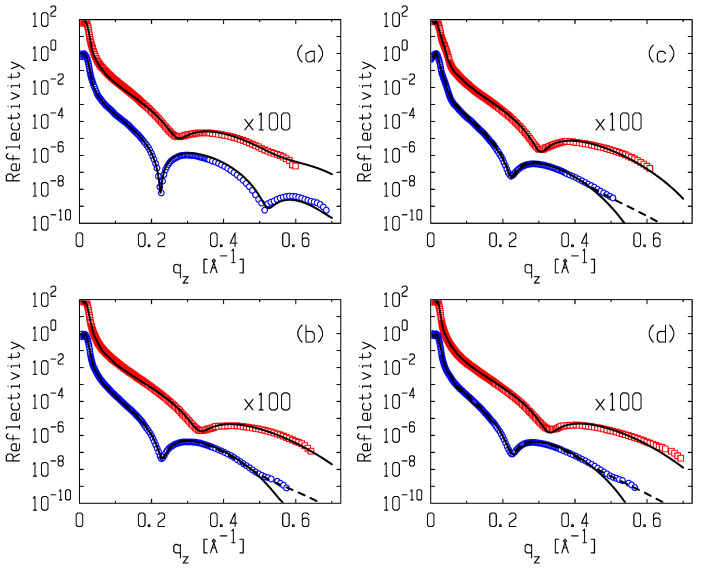
X-ray reflectivity, *R*(*q_z_*), for DMPS monolayers at the surface of water subphase and in the presence of polylysines at the area per lipid: A ≈ 40 Ǻ^2^ (blue circles; LC phase near the collapse) and A ≈ 70 Ǻ^2^ (red circles, LE phase). Panels correspond to the surface of the 10 mM KCl water subphase (**a**) and water solutions of PL-5 (**b**), PL-12 (**c**), and PL-200 (**d**), respectively. Error bars (in a range from 3% to 10%) representing the counting statistics for the datapoints are smaller than the symbols. Solid and dashed lines correspond to the model structures discussed in the text.

The scattering vector, **q** = **k**_in_ − **k**_sc_, at the specular reflection has only one nonzero component, $q_{z}=2k_{0}\sin\alpha$, where α is the glancing angle in the plane, normal to the surface. The value of the angle of the total external reflection for the surface of the water, *α_c_* ($q_{c}=2k_{0}\sin\alpha_{c}$), is fixed by the bulk electron density in it, *ρ*_w_ = 0.333 *e^−^*/Ǻ^3^, and the wavelength of photons, $\alpha_{c}=\lambda \sqrt{r_{e}\rho_{w}/\pi }\approx 0.017$, rad, where *r_e_* = 2.814 × 10^−5^ Ǻ is the classical radius of an electron. Reflection curves were measured for two values of area per molecule, *A*, in the phospholipid monolayer, *≅*40 Ǻ^2^ and ≅70 Ǻ^2^; those correspond to the LC and LE phases of the monolayer, respectively. The reflectivity curves for these systems are shown in Figure 4. The bulk scattering background was subtracted from the specular reflection data during the experiment. Error bars (in a range from 3% to 10%) representing the counting statistics for the datapoints in Figure 4 are smaller than the symbols.

An analysis of the reflectivity data was carried out within the framework of a model approach using the following expression for the reflection coefficient:

(1) $$R\left(q_{z}\right)\approx \frac{1}{\rho_{w}^{2}}{\left|\frac{q_{z}-q_{z}^{t}}{q_{z}+q_{z}^{t}}\right|}^{2}{\left|\sum_{j=0}^{N}\left(\rho_{j+1}-\rho_{j}\right)\exp\left(-i\sqrt{q_{z}q_{z}^{t}}\sum_{n=0}^{j}L_{n}\right)\exp\left(-\frac{1}{2}\sigma_{j}^{2}q_{z}q_{z}^{t}\right)\right|}^{2}$$

where $q_{z}^{t}=\sqrt{q_{z}^{2}-q_{c}^{2}}$. Next, a model profile, *ρ*(*z*), for the monolayer is constructed based on the error function, erf(x).

(2) $$\rho (z)=\frac{1}{2}\rho_{0}+\frac{1}{2}\sum_{j=0}^{N}\left(\rho_{j+1}-\rho_{j}\right)erf\left(\frac{l_{j}}{\sigma_{j}\sqrt{2}}\right),\;l_{j}(z)=z+\sum_{n=0}^{j}L_{n},\;erf(x)=\frac{2}{\sqrt{\pi }}\int_{0}^{x}e^{-y^{2}}dy$$

where $\rho_{N+1}\approx \rho_{w}$ is the electron concentration in the bulk of water, and $L_{0}\equiv 0$ is a position of the boundary between acyl tails and air (*z* = 0). Parameter $\rho_{0}\approx 0$ is the electron concentration in the air. All presented experimental dependencies of $R\left(q_{z}\right)$ can be described quite well within two-layer (*N* = 2) and three-layer models (*N* = 3). Expression (1) was obtained in DWBA (distorted wave born approximation), which relates *R* to the electron density gradient $d\langle \rho \left(z\right)\rangle /dz$ along the normal to the interface and averaged over it [31]. The approximation works quite well in the region of small angles of incidence, $\sigma q_{z}\leq 1$, but it is not entirely correct for $\sigma q_{z}>>1$.

## 4. Discussion

The structure of the water–lipid–air interfacial boundary can be conventionally represented as two layers (*N* = 2). In accordance with the structure of the DMPS molecule, the first layer of thickness, *L*_1_, and electron density, *ρ*_1_, are formed by aliphatic tails—C_14_H_27_. The second layer of thickness, *L*_2_, and the density, *ρ*_2_, are formed by polar groups of phosphatidylserine. In the experiment, thermal fluctuations (capillary waves) on the surface in the illumination region (~1 mm^2^) contribute to the observed structure, mainly leading to blurring the jumps in the model electron density profile [32]. While calculating curves (1) in Figure 4, we fixed the roughness parameters so that *σ*_0_ = *σ*_1_ = *σ*_2_. Their adjustable value, with good accuracy, turned out to be equal to the calculated value for the “capillary width”, $\sigma_{0}^{2}=\left(k_{B}T/2\pi \gamma \left(A\right)\right)\ln\left(Q_{\max}/Q_{\min}\right)$ (where $k_{B}$ is the Boltzmann constant), which is set by the short wavelength limit in the spectrum of capillary waves, $Q_{\max}=2\pi /a$ (*a* ~ 10 Ǻ in an order of magnitude equal to the intermolecular distance), and specified by the angular resolution, Δ*β* (≅0.023°), of the detector, $Q_{\min}=q_{z}^{\max}\Delta \beta$, in the experiment [33]. The same approach to the analysis of reflectometry data was also successfully used by us earlier, for example, to study structures and phase transitions in the adsorption films of amphiphilic molecules at both macroscopically planar oil–water interfaces and water surface [34,35].

**Figure 5:**
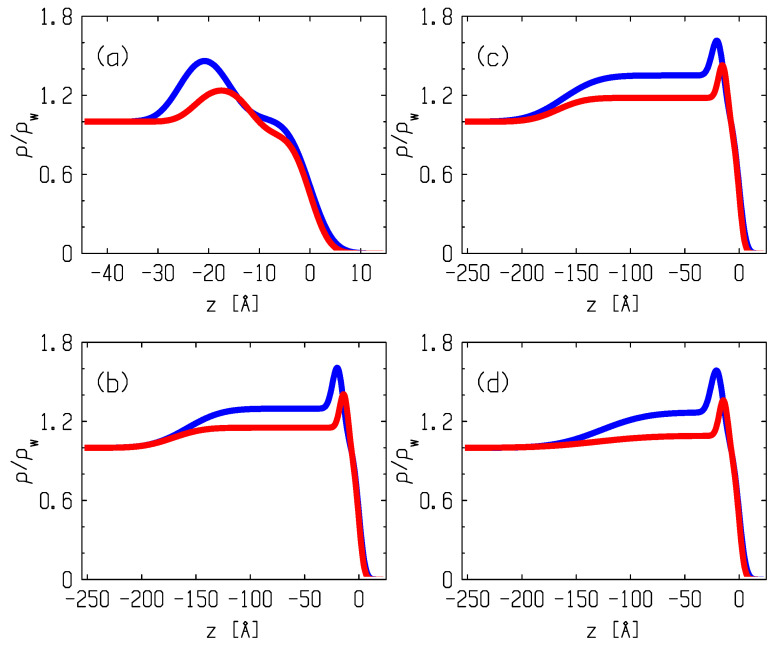
Surface–normal electron density profiles, *ρ*(z), normalized to the electron density in water under normal conditions, *ρ*_w_ = 0.333 *e^−^*/Ǻ^3^. Blue lines are for films at the condensed LC state of the lipid monolayer near the collapse, A≈ 40 Ǻ^2^. Red lines are for films at the liquid LE state of lipids below the phase transition at a value of area per molecule, A ≈ 70 Ǻ^2^. Panels correspond to the surface of the 10 mM KCl water subphase (**a**) and water solutions of PL-5 (**b**), PL-12 (**c**), and PL-200 (**d**), respectively.

The model profile for the pure monolayer (*N* = 2, *σ*_0_ = *σ*_1_ = *σ*_2_) is shown with curves in Figure 5a. Table 2 exhibits the values of the fitting parameters for DMPS monolayers with area per molecule, ~40 Ǻ^2^ and ~70 Ǻ^2^. These parameters of the DMPS monolayer in the LE and LC states are in agreement with our previous results [13]. The adsorption of the polymer is well traced by the change in the reflection curves for both states of the monolayer.

The three-layer model (*N* = 3) describes the phenomenon well. It is assumed that the packing parameters *ρ*_1_ and *L*_1_ of the aliphatic tail-packing of the phospholipid are equal to the parameters of the pure monolayer in the corresponding state. The calculated curves for $R\left(q_{z}\right)$ are shown in Figure 4b–d by solid lines, and the corresponding model profiles, $\rho \left(z\right)$, are presented in Figure 5b–d. Good agreement between the calculated curves and the experiment is achieved by fitting the parameters for the layer of the polar groups, *ρ*_2_ and *L*_2_ (constraints: *σ*_0_ = *σ*_1_ = *σ*_2_), and the parameters of the polymer film, namely, the thickness, *L_3_*; density, *ρ_3_*; and width, *σ*_3_, of the polymer film–subphase boundary (*σ*_3_ >> *σ*_0_).

At large angles of incidence (*q_z_* > 0.5 Ǻ^−1^), the fitting curves for the LC state exhibit a deviation from the reflectivity data. The discrepancy can be eliminated by canceling the strict condition *σ*_0_ = *σ*_1_ = *σ*_2_ in the fitting procedure, which indicates a more complex noncapillary-wave surface (or intrinsic) structure than the one considered above. The dashed lines in Figure 4 show the reflectivity curves calculated for polylysine–lipid films in the LC state under the new condition, *σ*_1_ = *σ*_2_. Equation (1) approximates *R(q_z_)* quite well for *q_z_* > 0.5 Ǻ^−1^ when *σ*_0_ ~ 4 Ǻ and *σ*_1_ = *σ*_2_ ~ 6 Ǻ. The remaining parameters of the equation, within the limits of errors, coincide with those given in Table 2. Thus, we estimate the width of the intrinsic structure associated with the lipid head group region to be as large as $\sqrt{\sigma_{1}^{2}-\sigma_{0}^{2}}$ ~ 5 Ǻ [21].

Recently, a theoretical analysis of electrokinetic measurements led us to the conclusion that the lateral distribution of large polycations never reaches a uniform surface coverage [36,37]. The contribution of heterogeneities (domains) in the polymer layer to *R*(*q_z_*) for most cases of “mosaic structure” is ambiguous, primarily due to averaging over the illumination area in the experiment [38]. In addition, signs of the intrinsic structure appear in the reflectivity at *q_z_* > 0.5 Ǻ^−1^, which is practically at the DWBA applicability limit (*σ_0_q_z_* > 2). Therefore, a comprehensive approach to determining the structure would be helpful, for example, by simultaneously measuring both reflectivity and off-specular scattering intensity, preferably using an X-ray beam with a higher spatial coherence length than in the current study (~100 Å) [39,40].

According to our analysis, with respect to polymer adsorption effects, firstly, the integral density of the layer of the polar groups, *Aρ_2_L_2_*, drops significantly (about two times) in the LE phase, whereas for LC, the drop in the density is not so significant (less than 10%). Secondly, the fitting parameter, *ρ_3_*, depends on the area per lipid, *A*, in the monolayer. In the LE states, *ρ*_3_ varies in a range of 1.1–1.2*ρ_w_*, while in the LC state, *ρ_3_ ≈* 1.3*ρ_w_*. The value *L*_3_ = 100–150 Ǻ weakly depends on the density of the monolayer. It follows that a twofold decrease in the area per molecule in the monolayer, from 70 Ǻ^2^ to 40 Ǻ^2^, leads to an approximately twofold increase in the near-surface density, *ρ_3_*, of the adsorption film of the macromolecules: $\left(\rho_{3}^{LC}-\rho_{w}\right)/\left(\rho_{3}^{LE}-\rho_{w}\right)$~ 2. Thirdly, the value of *σ*_3_ ~ 20–40 Ǻ significantly exceeds the capillary width, *σ*_0_, which indicates the diffuse nature of the structure of the polymer film–subphase interface. An estimate of the hydration of the polar region can be obtained using the following equation: $n=\left(1/\Gamma_{w}\right)\left(A\rho_{1}L_{1}+A\rho_{2}L_{2}-\Gamma \right)$, where Γ = 391 is the number of electrons in the DMPS molecule, and Γ*_w_* = 10 is the number of electrons in H_2_O [13].

The compressibility diagram and Volta potential are registered with the Langmuir monolayer technique and further analyzed within the empirical approach. This approach allows us to speculate about lipid molecule hydration sizes and their formal elasticity in the presence of polypeptides at the interface in the LE state and in the intermediate region. The most reliable results in this respect were found using synchrotron X-ray reflectometry applied to monolayer films with adsorbed polypeptides and analyzed by two- and three-layer models. According to Table 2, the geometrical parameters and degree of hydration of the DMPS monolayers at the surface of the buffer water are consistent with the results of our previous study of the LE-LC transition (~18 H_2_O molecules in the LE phase and ~3 H_2_O in the LC phase) [13]. However, the reflectivity data indicate significant structural changes in the monolayers in the presence of poly-D-lysine hydrobromide molecules at its water interface in both the LE and LC states. The three-layer model adequately describes reflectivity data in the entire *q_z_*-range with parameters reflecting the dehydration of lipid monolayers as a result of polypeptide adsorption. On the one hand, in the LE phase of the monolayer (area per molecule A ≅ 70 Ǻ^2^), the integrated electron density, *A*ρ_2_*L*_2_, of the lipid headgroup region is approximately half the density contained in the clean monolayer. This indicates both significant compaction and dehydration in the polar groups of the lipids caused by the adsorption of polypeptides. On the other hand, in the LC state (A ≅ 40 Ǻ^2^), the degree of the hydration effect is similar to that of the initial DMPS monolayer. However, both the electron density and the thickness of the head group region differ significantly from the values of these parameters for the clean monolayer. Finally, according to the fitting parameters in Table 2, both the thickness and density of the polymer layer of PL-200 are noticeably smaller than both PL-5 and PL-12. This may reflect the effect of inhomogeneity in the adsorption layer for large polylysine molecules, which follows from the theoretical analysis of the electrokinetic data [36,37].

## 5. Conclusions

The experimental data presented in our work demonstrate a significant change in the physicochemical characteristics of a DMPS monolayer upon the adsorption of polylysine molecules on its surface. According to previous studies [14,26], the maximum coverage of the surface by the polymers of different sizes occurs with approximately the same content in the aqueous subphase, expressed in terms of the concentration of polymer bases. Judging from the shape of the compression diagrams, the adsorption of these macromolecules on the surface of the monolayer retains the possibility of a phase transition in the phospholipids from the liquid–crystalline to the condensed state in approximately the same region of areas per lipid as the initial phospholipid monolayer. Based on the series of compression diagrams for pentalysine (Figure 1), a general trend can be seen, showing that, with an increase in the concentration of PL-5 in the aqueous subphase, an increase in the “rigidity” of the lipid monolayer occurs. This is also accompanied by a decrease in the range of the Volta potential variations. At high concentrations, when the surface of the monolayer is completely covered with this polymer, the observed value of "rigidity" approaches the same value for all polylysines studied.

It turns out that the binding of polymers to a DMPS monolayer leads to a smaller variation in the Volta potential in both the LE and LC states than for the initial clean monolayer (Figure 2). As a result, the overall change in voltage potential due to the compression of the DMPS monolayer is significantly reduced in the presence of the adsorbed polymer. The increment of the boundary potential became smaller since its dipole component partially compensates for the contribution of the positive charge of the adsorbed polycations. This fact correlates well with the previously mentioned data on the adsorption of lysine on the surface of planar lipid membranes [14].

More subtle effects associated with a change in the structure of the lipid membrane interfacial boundary in the presence of polylysines appear in the X-ray reflectivity. The application of the model approach implemented by us earlier for the analysis of X-ray data for such a system turned out to be useful if we consider interfacial structures in the form of two and three layers.

We assumed that both the thickness of the hydrophobic layer and the packing of hydrocarbon chains of the phospholipid do not change during the adsorption of the polylysines. Our analysis leads to several fairly reliable conclusions about the effect of polypeptides on the structure of the interface in the LE and LC states of the DMPS monolayer. First, our data reveal a systematically low integral electron density, *Aρ_2_L_2_*, in the polar region of the monolayer in the presence of the polymer layer compared with the pure monolayer. We interpret this to be a significant dehydration of the polar region of lipids, especially pronounced in the LE state, caused by polylysines. Note that our estimates for the parameters of the clean DMPS monolayers are in excellent agreement with the results of our earlier study [13]. Second, the densities of the polymer adsorption layer for all polylysines vary with the lateral density of lipids in the LE and LC phases of the monolayer (Figure 5). Third, the width of the polymer layer–water interface, >20 Ǻ, significantly exceeds the capillary width, *σ*_0_, indicating the diffuse blurring of the outer boundary of the layer facing the aqueous phase.

Finally, the presence of a polymer layer on the surface of a monolayer significantly increases the depth of the interfacial structure, which requires a comprehensive approach to determine the surface structure based on X-ray scattering data. To obtain more detailed information, both on the structure of the polymer layer and on the possible intrinsic structure of the head group region, an experiment with simultaneous measurements of reflectivity and off-specular scattering would be useful.

## Figures and Tables

**Table 1:** Fitting parameters for solid curves shown in Figure 1.

System/Parameters	*A*_0_*,* Ǻ^2^	*A_e_,* Ǻ^2^	*K_P_*, mN/m	t_0_, mN/m	*S*
DMPS (LE)	59.8	49.3	8.1	0.08	0.132
DMPS + PL-200 (LE)	60.8	48.9	13.6	0.07	0.057
DMPS (LC)	46	30.6	5.2	-	-
DMPS + PL-200 (LC)	49	24.4	7.0	-	-

**Table 2:** Fitting parameters for fits with Equation (1) to the X-ray reflectivity from the DMPS monolayers with the area per lipid molecule, A. *σ*_0_ is the width of the acyl group layer–air interface. *L*_1_ and *L*_2_ are the thicknesses of the acyl group layer with the density *ρ*_1_ and the headgroup regions with the density *ρ*_2_, respectively. *L*_3_ and *ρ*_3_ are the thickness and electron density of the polymer layer, correspondingly. *σ*_3_ is the roughness parameter for the water–polymer layer interface. *n* is the estimated number of hydrated water molecules per lipid molecule obtained from the two-layer model for the lipid layer. The electron densities are normalized to the electron concentration in water under normal conditions, *ρ*_w_ = 0.333 *e^−^*/Ǻ^3^. The error bars were estimated utilizing the conventional χ^2^ criteria at a confidence level of 0.95.

	Hydrophobic Tail Region			Headgroup Region			Polymer Layer		
	*L*_1_ (Å)	$\rho_{1}/\rho_{w}$	*σ_0_* (Å)	*L*_2_ (Å)	$\rho_{2}/\rho_{w}$	*n*	*L*_3_ (Å)	$\rho_{3}/\rho_{w}$	*σ*_3_ (Å)
	*A* ≈ 40 Å^2^								
Water	16.1 ± 0.4	1.02 ± 0.01	3.6 ± 0.2	9.5 ± 1	1.56 ± 0.01	3 ± 1			
PL-5			4.6 ± 0.2	6.0 ± 0.5	2.1 ± 0.1	0 ± 1	144 ± 3	1.29 ± 0.02	40 ± 3
PL-12			5.0 ± 0.2	6.0 ± 0.5	2.1 ± 0.1	0 ± 1	112 ± 3	1.29 ± 0.04	25 ± 2
PL-200			4.9 ± 0.2	6.9 ± 0.5	2.0 ± 0.1	1 ± 1	103 ± 3	1.27 ± 0.02	33 ± 3
	*A* ≈ 70 Å^2^								
Water	12 ± 0.3	0.90 ± 0.02	3.0 ± 0.2	11 ± 1	1.25 ± 0.04	18 ± 3			
PL-5			3.9 ± 0.2	2.5 ± 0.5	2.5 ± 0.5	1 ± 1	167 ± 5	1.21 ± 0.02	29 ± 3
PL-12			3.6 ± 0.2	5.2 ± 0.4	1.5 ± 0.1	4 ± 1	165 ± 5	1.18 ± 0.04	19 ± 2
PL-200			3.9 ± 0.2	3.4 ± 0.5	2.1 ± 0.5	3 ± 1	115 ± 3	1.09 ± 0.02	41 ± 3

## Data Availability

The data presented in this study are available on request from the corresponding authors.
